# Venous ectasia preceding intra-tumoral hemorrhage in a case of gliosarcoma with transverse sinus involvement

**DOI:** 10.1093/jscr/rjad429

**Published:** 2023-07-29

**Authors:** Chaejin Lee, Mee-Seon Kim, Jeong-Hyun Hwang, Seong-Hyun Park, Ki-Su Park, Sang-Youl Yoon

**Affiliations:** Department of Neurological Surgery, Asan Medical Center, University of Ulsan College of Medicine, Seoul, Korea; Department of Pathology, School of Medicine, Kyungpook National University Hospital, Kyungpook National University, Daegu, Korea; Department of Neurosurgery, School of Medicine, Kyungpook National University Hospital, Kyungpook National University, Daegu, Korea; Department of Neurosurgery, School of Medicine, Kyungpook National University Hospital, Kyungpook National University, Daegu, Korea; Department of Neurosurgery, School of Medicine, Kyungpook National University Chilgok Hospital, Kyungpook National University, Daegu, Korea; Department of Neurosurgery, School of Medicine, Kyungpook National University Chilgok Hospital, Kyungpook National University, Daegu, Korea

## Abstract

Although intratumoral hemorrhage is common in patients with malignant brain tumors, reports on its clinical course are scarce. This report presents a rare case of a patient with intratumoral hemorrhage with gliosarcoma invading the venus sinus. This invasion and a small draining vein were observed at diagnosis. Magnetic resonance imaging performed 1 week later showed new-onset venous ectasia, which caused intratumoral hemorrhage. This case provides insight into the mechanisms underlying intratumoral hemorrhage and highlights the emergence of new intratumoral vasculature as a potential warning sign for hemorrhage.

## INTRODUCTION

Gliosarcomas, accounting for ~2–8% of glioblastomas, are characterized by a more aggressive course compared with other glioblastomas [1]. They are isocitrate dehydrogenase (IDH) wild-type glioblastomas characterized by a biphasic tissue pattern of sarcomatous and gliomatous areas. Compared with other glioblastoma subtypes, gliosarcomas containing more sarcomatous components and exhibiting more severe angioinvasive or angiosarcomatous features are considered to be at a greater risk of intratumoral bleeding [2–4]. The presence of abnormal vascular structures is common in tumoral tissue, although low-flow angiostructures associated with venous ectasia rarely increase the risk of intratumoral hemorrhage. The current report presents a case of a patient who had intratumoral bleeding and gliosarcoma without angiosarcomatous features invading the transverse sinus.

## CASE REPORT

**Figure 1:**
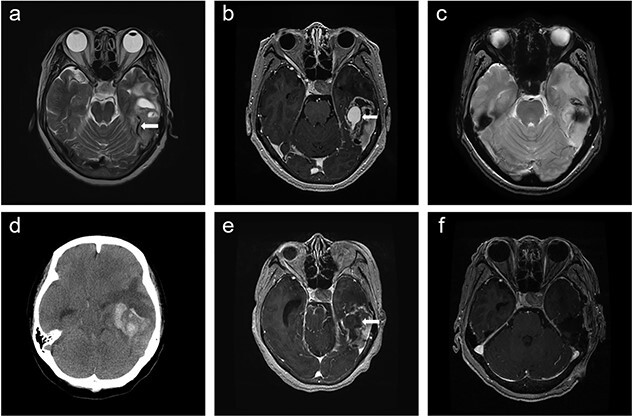
Time course of brain images during intratumoral hemorrhage formation; (**a**) initial T2-weighted image showing the tumor-draining vein without venous ectasia (arrow); (**b**, **c**) T1-enhanced image and gradient-echo image obtained 1 week after the initial MRI showing new-onset venous ectasia (arrow) without internal thrombus; (**d**, **e**) non-contrast-enhanced brain CT and T1-enhanced images obtained 1 day after images; (b) and (c) showing intratumoral hemorrhage with an intra-luminal thrombus (arrow); (**f**) post-operative T1-enhanced image.

A 66-year-old woman presented with progressive cognitive deterioration and dysarthria. Non-contrast-enhanced brain magnetic resonance imaging (MRI) showed a 6-cm intraaxial mass in the left temporal lobe abutting the dura and an incidentally identified pituitary mass (Fig. 1a).

The patient was diagnosed with cerebral ischemic stroke and atrial fibrillation 2 years ago. Since then, she had been taking edoxaban (60 mg/day). She was scheduled to discontinue edoxaban from the day of hospitalization and to undergo brain tumor surgery 4 days later.

Physical examination revealed global dysphagia accompanied by mild cognitive decline. The results of preoperative laboratory and coagulation tests were unremarkable. Gadolinium-enhanced brain MRI revealed lesion in the left temporal lobe exhibiting an irregular ring-enhancing component with central necrosis. Follow-up MRI showed new-onset venous ectasia of the tumor-draining vein into the transverse sinus, which was not observed on initial MRI (Fig. 1b). The draining vein joined the transverse sinus and narrowed at the confluence point. Gradient echo imaging showed that the lumen of the vein appeared patent without internal thrombus (Fig. 1c).

The next day, the patient experienced sudden headache and rapid consciousness deterioration. Brain computed tomography (CT) revealed acute hemorrhage inside the tumor with a midline shift (Fig. 1d). Navigational MRI showed that the contrast agent failed to fill the lumen of the venous dilatation (Fig. 1e).

**Figure 2:**
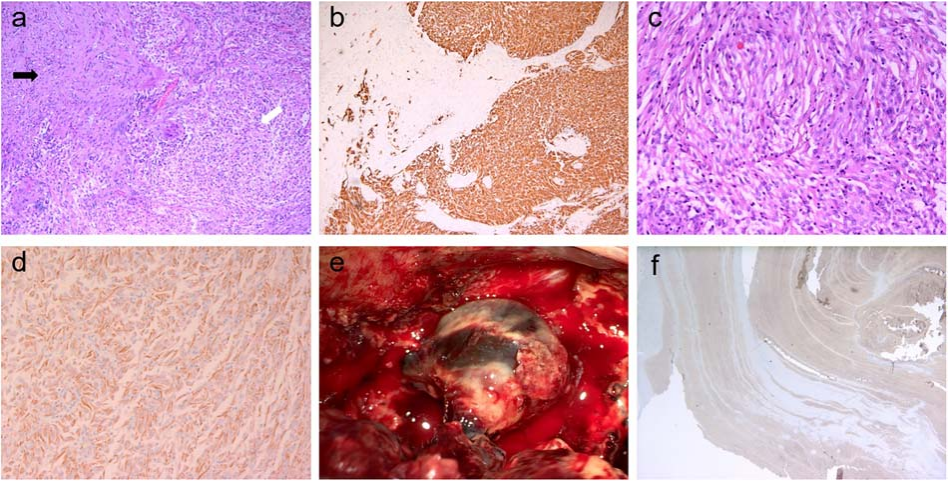
Pathologic images of the tumor and thrombosed venous ectasia; (**a**) the tumor had a biphasic appearance (white arrow: glial component, black arrow: sarcomatous component); the glial component had features of typical glioblastoma with nuclear pleomorphism and microvascular proliferation as well as positive immunoreactivity for glial fibrillary acidic protein (**b**); the sarcomatous area (**c**) contained spindle cells with moderate nuclear atypia and exhibited diffuse positivity for vimentin (**d**) protein; intraoperative view of thrombosed venous ectasia is presented in (**e**); histological analysis revealed that the vessel wall was negative for actin (**f**).

Intraoperatively, the tumor exhibited soft and friable consistency along with the presence of hematoma and cystic region filled with yellowish necrotic material. The tumor invaded the transverse sinus lumen and was firmly attached to the adjacent dura. The venous ectasia was confirmed to be the tumor-draining vein with internal thrombosis (Fig. 2e). After surgery, the patient was treated with concurrent chemoradiotherapy (CCRT). Brain MRI performed after CCRT showed no signs of disease progression, and the patient underwent adjuvant chemotherapy without any neurological deterioration.

Pathological examination revealed an IDH wild-type, World Health Organization Grade 4 gliosarcoma. The tumor cells exhibited immunoreactivity for glial fibrillary acidic protein and vimentin in the glial and sarcomatous components, respectively. The glial component had features of a typical glioblastoma with nuclear pleomorphism and microvascular proliferation. Contrarily, the sarcomatous component contained spindle cells with moderate nuclear atypia. Histological examinations were conducted separately for thrombosed venous ectasia. The immunohistochemical results for actin in the vessel wall were negative, suggesting that the vessel wall had developed within a short period without forming a normal vascular structure (Fig. 2).

## DISCUSSION

Several studies have demonstrated rapid tumor progression accompanied by abnormal angiogenesis and tumor invading large vessels in patients with highly malignant tumors, and both are associated with an increased risk of intratumoral hemorrhage [5, 6]. Previous studies have reported a significant upregulation in the expression levels of the molecular markers of vascular endothelial and basic fibroblast growth factors within glioblastoma tissues with intratumoral hemorrhage compared with those without such hemorrhagic manifestations [7, 8]. Research has further suggested that bleeding risk increases with the development of small vessels near the necrotic area (i.e. neoangiogenesis) [9].

In addition to tumor inherent malignancy, tumor location is assumed to be associated with an increased susceptibility to intratumoral hemorrhage. Hersh *et al*. reported a case of chondromyxoid fibroma invading the transverse-sigmoid sinus junction exhibiting cerebellar hemorrhage [10]. Sumi *et al*. presented a case of meningioma invading the sigmoid sinus, which resulted in intracranial hypertension due to venous hypertension [11]. Both cases involved low-grade solid, fibrous tumors, which potentially caused venous outflow obstruction or severe stenosis of the venous sinus. Our patient was diagnosed with gliosarcoma and had solid mass with a sarcomatous component and adherence to the dura mater. These characteristics potentially contributed to venous obstruction. Unlike most benign tumors invading the venous sinus without causing symptoms due to adequate venous collaterals, gliosarcomas have rapid growth, which potentially caused the intratumoral hemorrhage in this case.

Our patient had a structural change in the intratumoral traversing vessel within a mere week, subsequently leading to rupture of the lesion and necessitating emergent surgical intervention. This phenomenon provides insight into the mechanisms underlying intratumoral hemorrhage in gliosarcoma cases. The initial brain MRI showed a gradual narrowing of the tumor-draining vein that joined the left transverse sinus. Hence, we considered that tumor invasion into the left transverse sinus obstructed venous flow, consequently inducing venous stenosis. Brain MRI performed 1 week after the initial MRI revealed new-onset venous ectasia without thrombus. During this examination, the signal void demonstrated reduced flow despite the lumen being open. Just one day later, we observed an abrupt occurrence of intratumoral hemorrhage accompanied by thrombus formation within the venous ectasia. These imaging results suggest that the structural changes in the venous system were directly associated with the intratumoral hemorrhage. Tumor invasion into the venous sinuses disrupts blood flow, leading to flow stasis and subsequent development of proximal venous ectasia. Ectasia is further complicated by thrombus formation within its lumen. These vascular changes, coupled with an increase in arteriovenous pressure in the tumor vessels, ultimately culminate in the manifestation of intratumoral hemorrhage. Notably, the rapidly proliferating nature of gliosarcomas emphasizes the ability of such vascular structural alterations to occur within a remarkably short timeframe, as demonstrated that our patient.

To our knowledge, this is the first report of gliosarcoma with intratumoral hemorrhage and new-onset venous ectasia. Details of the present case emphasize the need to consider structural changes in low-flow vascular malformations, such as venous ectasia, as warning signs of intratumoral hemorrhage. Furthermore, clinicians should be aware of the increased risk of intratumoral hemorrhage in patients with rapidly growing tumors invading the venous sinus due to dramatic vascular flow changes.

## Data Availability

The datasets generated and/or analyzed during the current study are available from the corresponding author on reasonable request.
